# The TNFalpha gene relates to clinical phenotype in alpha-1-antitrypsin deficiency

**DOI:** 10.1186/1465-9921-9-52

**Published:** 2008-07-11

**Authors:** Alice M Wood, Matthew J Simmonds, Darren L Bayley, Paul R Newby, Stephen C Gough, Robert A Stockley

**Affiliations:** 1 Division of Medical Sciences, The Medical School, University of Birmingham, Birmingham, B15 2TT, UK; 2 Lung Investigation Unit, University Hospital Birmingham, Birmingham, B15 2TH, UK

## Abstract

**Background:**

Genetic variation may underlie phenotypic variation in chronic obstructive pulmonary disease (COPD) in subjects with and without alpha 1 antitrypsin deficiency (AATD). Genotype specific sub-phenotypes are likely and may underlie the poor replication of previous genetic studies. This study investigated subjects with AATD to determine the relationship between specific phenotypes and *TNFα *polymorphisms.

**Methods:**

424 unrelated subjects of the PiZZ genotype were assessed for history of chronic bronchitis, impairment of lung function and radiological presence of emphysema and bronchiectasis. A subset of subjects with 3 years consecutive lung function data was assessed for decline of lung function. Four single nucleotide polymorphisms (SNPs) tagging *TNFα *were genotyped using TaqMan^® ^genotyping technologies and compared between subjects affected by each phenotype and those unaffected. Plasma TNFα levels were measured in all PiZZ subjects.

**Results:**

All SNPs were in Hardy-Weinberg equilibrium. A significant difference in rs361525 genotype (p = 0.01) and allele (p = 0.01) frequency was seen between subjects with and without chronic bronchitis, independent of the presence of other phenotypes. TNFα plasma level showed no phenotypic or genotypic associations.

**Conclusion:**

Variation in *TNFα *is associated with chronic bronchitis in AATD.

## Background

Chronic obstructive pulmonary disease (COPD) is thought to be the result of environmental triggers in genetically susceptible individuals. Although cigarette smoking is the main environmental risk factor, only about 15% of smokers develop clinically significant disease[1], suggesting other influences on disease expression. This is supported by family studies showing ancestral aggregation of spirometric abnormalities both in the general population [2] and in relatives of patients with COPD [3]. Moreover, differences in rate of decline of lung function between smokers[4] suggests a gene-environment interaction.

COPD is associated with an abnormal inflammatory response in which proteases[5] and oxidants[6] play a significant pathogenic role. Inflammation is particularly important in systemic disease and associated co-morbidities[7]. Tumour necrosis factor alpha (TNFα) is an inflammatory cytokine which is elevated in the sputum[8], bronchial biopsies[9] and circulation [10] of COPD patients. The TNFα gene (*TNFα*) has been investigated in several COPD phenotypes [11-14] but in general associations have been poorly replicated[13].

In AATD there is significant variation in the clinical phenotype. Although classically associated with basal pan-lobular emphysema, it is now recognised that multiple phenotypes typical of usual COPD occur, including centrilobular emphysema, bronchitis and bronchiectasis [15]. As in usual COPD other genetic influences may be important in determining clinical features [16-19], but no studies have examined the role of candidate genes in the full range of COPD phenotypes. Since inflammation is more marked in AATD than usual COPD [20], perhaps driven by a key interaction between AAT and TNFα [21], we hypothesised that variation in *TNFα *may influence clinical phenotype in AATD.

## Methods

### Study design

In this case-control association study, cases comprised subjects with AATD affected by a COPD phenotype and those unaffected acted as AATD controls. Subjects were genotyped for four tag single nucleotide polymorphisms (SNPs) selected to cover common variation in *TNFα*. Genotype and allele frequency were compared between cases and controls, with adjustment for confounding variables and phenotypic overlap. Plasma TNFα levels were compared with SNP genotype.

### Study subjects

Four hundred and twenty four unrelated Caucasian subjects from the UK national registry for AATD were studied. Ethical approval was given by the local ethics committee. All patients had a serum alpha-1-antitrypsin (AAT) level of <11 μM and PiZZ genotype confirmed by specific PCR (Heredilab, Salt Lake City, USA). None of the subjects had ever received AAT replacement.

### Clinical phenotyping

A full clinical assessment including smoke exposure, presence of chronic bronchitis (defined as a productive cough for at least 3 months of at least 2 consecutive years[22]), lung function testing and high resolution CT scanning of the chest was undertaken, as described previously [23]. Annual lung function measurement allowed assessment of decline of FEV1. Linear regression was used to calculate decline over 3 years, such that the slope of the line between time zero and 3 years for each subject represented loss per year. The presence of bronchiectasis was determined as described by Naidich *et al *[24] by a single radiologist. The presence of emphysema was determined by the appearance of the scan and density mask analysis of slices at the level of the aortic arch (representing the upper zone) and the inferior pulmonary vein (representing the lower zone) using a threshold of -910HU. This threshold has been deemed optimal for emphysema detection[25], and validated against physiological measures in AATD [26]. Patients whose voxel index exceeded values seen in normal subjects in either zone[27] were deemed to have emphysema.

### Genotyping and TNFα quantification

SNPs to tag *TNFα *were chosen using linkage disequilibrium (LD) data for UK Caucasians [28], according to established methods [29], revealing four tags needed to capture all SNPs with minor allele frequency >0.05 using a cut off of r^2^>0.8. DNA extraction was performed using a modified Nucleon Bacc II kit (Tepnel Life Sciences, UK) and quantified using Picogreen^® ^(Molecular Probes Inc, UK). Genotyping was carried out using TaqMan^® ^genotyping technologies (Applied Biosystems, UK) on an ABI7900 HT. All genotyping assays were pre-validated by the suppliers, and all plates included appropriate negative controls. Stable state plasma TNFα was measured using a high sensitivity ELISA (R & D systems, Abingdon, UK) as described previously [30].

### Statistical Analysis

Deviation from Hardy Weinberg equilibrium was checked for all SNPs. Data was analysed using SPSS (version 12, November 2003, Chicago: SPSS Inc) and haplotype analyses carried out in Haploview[31]. Comparisons of demographic and clinical features between subgroups were carried out using the Mann Whitney or t-tests, as appropriate. Genotype and allele frequency of each tag SNP was compared between AATD subjects with and without emphysema, bronchiectasis and chronic bronchitis using logistic regression controlling for age, gender, smoke exposure and presence of additional phenotypes. Qualitative outcomes were chosen where possible since they may be more informative in AATD[32]. Quantitative genetic association analysis was carried out for FEV1, FEV1/FVC, KCO, upper zone voxel index (UZVI) and lower zone voxel index (LZVI) using general linear models, accounting for covariates as before. Analyses of HRCT measures were only carried out in those with emphysema. Additive models were assumed for all SNPs. Plasma TNFα level was compared between genotypes for each associated SNP using regression controlling for age; no other covariates were significant in the models.

## Results

### Clinical phenotypes

**Figure 1:**
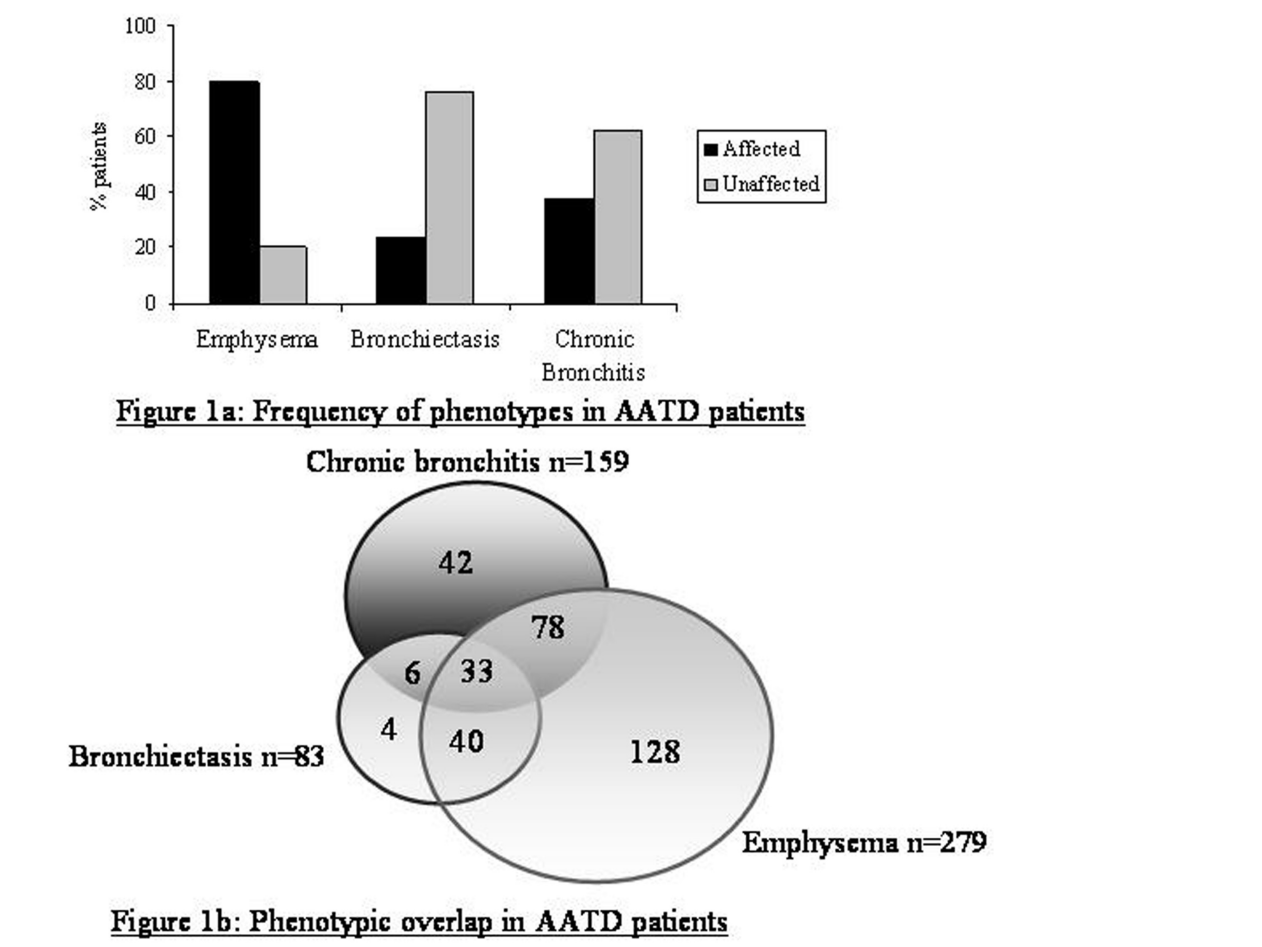
text

The clinical features of the whole group and of those with each sub-phenotype are shown in table 1. The frequency of each qualitative phenotype within the subjects is shown in figure 1a, and the degree of overlap between them in figure 1b. Emphysematous subjects had smoked more than those without (p < 0.001). There was no difference in smoke exposure, lung function or UZVI between those with and without bronchiectasis, though affected subjects were older (p < 0.001) and exhibited more severe lower zone emphysema (p = 0.03). Subjects with chronic bronchitis had lower FEV1 (p = 0.01) but no other demographic or clinical features differed. There were no gender differences between those with and without each of the qualitative phenotypes. The group assessed for decline of lung function showed no difference in any demographic or clinical features from the whole group, except that they had smoked slightly more (p = 0.030).

**Table 1:** Clinical features of the patient group.

	Whole group n = 424	Decline group n = 110	Emphysema		Bronchiectasis		Chronic bronchitis	
			
			Yes n = 279	No n = 70	Yes n = 83	No n = 266	Yes n = 159	No n = 265
Gender (% male)	59.91	66.36	64.16	54.29	57.83	63.67	64.78	57.03
Age (years)	50.01 (0.52)	51.48 (0.92)	51.76 (0.78)	41.52 (33.77–49.27)	54.18 (1.03)	48.90 (0.63)	50.79 (0.77)	49.48 (0.69)
Pack years	14.00 (2.35–25.65)	18.75 (9.01–28.49)	17.50 (7.65–27.35)	0 (0–4.18)	14.63 (4.33–25.93)	13.50 (1.40–24.60)	14.63 (2.55–26.71)	12.75 (0.75–24.75)
FEV1 %predicted	37.15 (18.30–56.04)	35.01 (18.25–51.77)	32.08 (18.94–45.22)	102.88 (80.93–124.83)	36.64 (14.59–58.69)	35.54 (17.39–53.69)	32.76 (15.49–50.03)	38.17 (14.18–62.16)
FEV1/FVC	38.40 (25.95–50.85)	37.30 (27.10–48.40)	35.00 (26.00–44.00)	73.60 (63.10–84.10)	40.10 (24.35–55.85)	37.50 (25.00–48.00)	37.85 (26.85–48.85)	39.40 (26.40–52.40)
KCO %predicted	69.82 (1.18)	68.09 (2.12)	63.18 (1.38)	98.66 (2.55)	67.85 (2.75)	70.57 (1.54)	67.62 (2.31)	71.34 (1.89)
UZVI	31.09 (1.05)	32.84 (1.69)	32.71 (1.02)	2.31 (0.39)	33.45 (1.89)	30.63 (1.27)	32.98 (1.69)	29.88 (1.36)
LZVI	44.59 (1.21)	52.10 (38.53–65.68)	46.78 (1.14)	5.85 (1.01)	54.15 (39.50–68.80)	47.95 (32.15–63.75)	48.85 (34.60–63.10)	49.50 (31.90–67.10)
Decline of FEV1 (ml/yr)		31.3 (4.1)						

Data is presented as mean (SEM) or median (IQR) dependent on its distribution. UZVI = upper zone voxel index, LZVI = lower zone voxel index. The only significant difference between the decline group and the whole dataset was that they had smoked more (p = 0.01).

In the regression models age remained a significant predictor of both emphysema and bronchiectasis (B = 0.09 and 0.05 respectively, both p < 0.0001), whilst pack years smoked was associated with emphysema (B = 0.10, p < 0.0001). No demographic or clinical features predicted the development of chronic bronchitis.

### Genetic associations

Genotyping was successful in 95.3% of cases. All SNPs were in Hardy Weinberg equilibrium. Allele frequencies for each tag SNP are shown in table 2. A significant difference in allele frequency was seen between subjects with and without chronic bronchitis for rs361525 (both p = 0.01), with the A allele conferring an odds ratio (OR) of 2.08 (95% confidence interval 1.18–3.67) in the regression analysis. In the regression analyses for presence of emphysema and bronchiectasis no tag SNP made a significant contribution. There were no tag SNP associations with lung function, emphysema severity or decline of FEV1. No haplotypes were observed for analysis.

**Table 2:** Allele frequencies of tag SNPs.

	Minor Allele frequency (%) (OR, p value)			
SNP [major allele, minor allele]	All	Chronic bronchitis	Emphysema	Bronchiectasis

rs1800629 [G, A]	21.73	24.32	21.97	26.58
rs361525 [G, A]	8.60	11.76 (2.08, 0.01)	8.24	5.00
rs1799964 [T, C]	25.32	26.69	25.10	26.58
rs3093662 [A, G]	8.91	11.33	8.17	4.55

Odds ratio (OR) and p value are given in parentheses for significant associations only.

### Plasma TNFα associations

Plasma TNFα was significantly correlated with age (r = 0.21, p = 0.05), but did not show any variation between sub-phenotypes or correlation with disease severity as measured by lung function or HRCT. A regression model controlling for age did not show any association of plasma level with rs361525 genotype, either in the whole group or the subgroup with chronic bronchitis. Levels in each sub-phenotype are shown in table 3.

**Table 3:** Plasma TNFα levels in AATD subjects.

	Plasma TNFα (pM)
All subjects	0.074 (0.004)
Emphysema	0.079 (0.005)
Bronchiectasis	0.089 (0.012)
Chronic bronchitis	0.078 (0.007)

Data is expressed as mean (SEM).

## Discussion

Our results show an association between the A allele of rs361525 subjects and chronic bronchitis in AATD. One previous study in COPD genetics has tagged *TNFα*[13] but did not find an association with rs361525. Subjects included a family cohort with airflow obstruction, and cases with emphysema. No previous studies in COPD have looked for association of chronic bronchitis with rs361525.

The importance of chronic bronchitis has been recognised by other COPD genetic association studies [14,33]. Its definition has been standardised for many years in respiratory medicine[22], but remains a historical definition, thus prone to problems with patient recall. Nevertheless it is an important phenotype to ascertain, as it may affect patient management: mucolytics may be more appropriate in the presence of a productive cough[34], and co-existence with an HRCT diagnostic of bronchiectasis might prompt addition of airway clearance measures. A high incidence of HRCT changes suggestive of bronchiectasis is seen in patients with emphysema[35] and it is not yet clear whether all such subjects should be managed as aggressively as those with typical symptomatology. Anti-TNF therapy has been disappointing in COPD[36], but was not assessed specifically in chronic bronchitis despite the importance of TNFα in the pathophysiology of the condition[9]. Furthermore, response to anti-TNF treatment may be dictated by *TNFα *genotype[37], perhaps indicating a need for further studies directed to sub-phenotype or genotype.

Although there is a degree of overlap of COPD phenotypes, our results are specific to chronic bronchitis, and concur with prior evidence regarding a role for TNFα in airways disease [9]. Such an association is lacking for presence or severity of parenchymal disease, defined by emphysema on HRCT. This suggests different genetic influences upon the development of each sub-phenotype and supports the view that inadequate patient characterisation may underlie poor replication of previous COPD genetic association studies. It is also of interest that the influence of age and smoke exposure differed between COPD phenotypes in the regression models, further emphasising differences in pathogenesis. Some markers of disease, notably CT densitometry, can become abnormal at or below the age of 20 in AATD[38], such that it was important for us to include all adult PiZZ subjects in our analyses. Despite maximising our numbers in this way we were unable to show any *TNFα *effect on quantitative phenotypes, again emphasising the specificity of association with chronic bronchitis.

Our reported genetic association in AATD may be explained by the interaction between TNFα and AAT. TNFα release is suppressed by AAT in vitro[21] and in animal models[39]. Consequently, TNFα effects may be more pronounced in AATD where this suppression is reduced. These epistatic effects resulting from cytokine interactions have previously been reported in asthma in which multiple polymorphisms in genes involved in the Th2 immune response, rather than individual SNPs appear to increase the risk of disease [40].

rs361525 (G-238A) is located in the promoter region of *TNFα *and has been studied extensively in many different disease states, although little evidence exists for a functional effect. Cellular studies of *TNFα *promoter region SNPs suggest that G-238A increases gene transcription [41]. Conversely gene expression in patients with psoriasis (a disease with which the -238 A allele is strongly associated[42]) appears reduced [43]. Further functional work clarifying the role of rs361525 would be indicated if fine mapping of the region surrounding it confirmed it to be the functional variant associated with disease, rather than merely a polymorphism in linkage disequilibrium. Such studies are expensive, and alternative surrogate support for a functional effect may be gained by assessing plasma TNFα levels [44]. In the current study no association was found between rs361525 genotype and plasma TNFα levels. This may be because of the specific association with airways disease since airway and plasma inflammatory markers do not correlate in COPD [45]. However many factors influence airway inflammation in COPD, including bacterial colonisation[46] and cachexia [10] so any study of genetic association with airway inflammatory markers would require matching of subjects for these features.

The *TNFα *promoter region is in strong LD with HLA class II alleles in Caucasians [47], particularly between rs1800629 and the HLA DR3 haplotype. This has not been observed for rs361525, therefore it is unlikely that our results are due to LD with HLA alleles.

The results reported in our study are without adjustment for multiple statistical testing as corrections such as Bonferroni appear overly conservative in genetic association studies [48], hence are inappropriate. This is particularly true when several theories are being tested, as in our study in which we hypothesised that each SNP could be driving any of three qualitative COPD phenotypes, or affecting disease severity via several quantitative phenotypes. Statistical significance has been proposed if it reaches the level needed for a genome wide study (generally considered to be p ≤ 5 × 10^-5 ^[48]), or by replication in an independent population. We have considered the latter approach: a second small familial dataset does exist in the UK that could act as a replication group. However, within this dataset there is no difference in the prevalence of chronic bronchitis between family members [49]. As such we would be unable to test our hypothesis in this cohort. These challenges are inherent in studying AATD, as recruitment of datasets powered for genome wide significance using healthy AATD controls may be impossible, because of the high rates of disease phenotypes (see Figure 1a). International collaborations to create larger datasets could certainly help overcome this problem although geographical variation of genes, particularly those within the HLA region such as *TNFα*, may be a further confounding factor [50].

## Conclusion

The current study conducted on the UK national registry of AATD presents data to indicate that variation in *TNFα *influences phenotype in AATD. It further supports the hypothesis that the range of COPD phenotypes may be driven by specific underlying genetic susceptibilities in a similar manner to usual COPD.

## Competing interests

The authors declare that they have no competing interests.

## Authors' contributions

AMW assessed clinical phenotypes, conducted laboratory procedures, analysed data and drafted the manuscript. PRN and DLB assisted and supervised laboratory procedures. MJS, SCG and RAS supervised the project and reviewed the manuscript.

## References

[B1] ATS (1996). Cigarette smoking and health.. Am J Respir Crit Care Med.

[B2] Lewitter FI, Tager IB, McGue M, Tishler PV, Speizer FE (1984). Genetic and environmental determinants of level of pulmonary function. Am J Epidemiol.

[B3] Kueppers F, Miller RD, Gordon H, Hepper NG, Offord K (1977). Familial prevalence of chronic obstructive pulmonary disease in a matched pair study. Am J Med.

[B4] Fletcher C, Peto R (1977). The natural history of chronic airflow obstruction. Br Med J.

[B5] Stockley RA (1999). Neutrophils and protease/antiprotease imbalance. Am J Respir Crit Care Med.

[B6] MacNee W (2000). Oxidants/antioxidants and COPD. Chest.

[B7] Sevenoaks MJ, Stockley RA (2006). Chronic Obstructive Pulmonary Disease, inflammation and co-morbidity--a common inflammatory phenotype?. Respir Res.

[B8] Keatings VM, Collins PD, Scott DM, Barnes PJ (1996). Differences in interleukin-8 and tumor necrosis factor-alpha in induced sputum from patients with chronic obstructive pulmonary disease or asthma. Am J Respir Crit Care Med.

[B9] Mueller R, Chanez P, Campbell AM, Bousquet J, Heusser C, Bullock GR (1996). Different cytokine patterns in bronchial biopsies in asthma and chronic bronchitis. Respir Med.

[B10] Di Francia M, Barbier D, Mege JL, Orehek J (1994). Tumor necrosis factor-alpha levels and weight loss in chronic obstructive pulmonary disease. Am J Respir Crit Care Med.

[B11] Sakao S, Tatsumi K, Igari H, Shino Y, Shirasawa H, Kuriyama T (2001). Association of tumor necrosis factor alpha gene promoter polymorphism with the presence of chronic obstructive pulmonary disease. Am J Respir Crit Care Med.

[B12] Sakao S, Tatsumi K, Igari H, Watanabe R, Shino Y, Shirasawa H, Kuriyama T (2002). Association of tumor necrosis factor-alpha gene promoter polymorphism with low attenuation areas on high-resolution CT in patients with COPD. Chest.

[B13] Hersh CP, Demeo DL, Lange C, Litonjua AA, Reilly JJ, Kwiatkowski D, Laird N, Sylvia JS, Sparrow D, Speizer FE, Weiss ST, Silverman EK (2005). Attempted replication of reported chronic obstructive pulmonary disease candidate gene associations. Am J Respir Cell Mol Biol.

[B14] Huang SL, Su CH, Chang SC (1997). Tumor necrosis factor-alpha gene polymorphism in chronic bronchitis. Am J Respir Crit Care Med.

[B15] Needham M, Stockley RA (2004). Alpha 1-antitrypsin deficiency. 3: Clinical manifestations and natural history. Thorax.

[B16] Rodriguez F, de la Roza C, Jardi R, Schaper M, Vidal R, Miravitlles M (2005). Glutathione S-transferase P1 and lung function in patients with alpha1-antitrypsin deficiency and COPD. Chest.

[B17] Silverman EK, Province MA, Campbell EJ, Pierce JA, Rao DC (1990). Variability of pulmonary function in alpha-1-antitrypsin deficiency: residual family resemblance beyond the effect of the Pi locus. Hum Hered.

[B18] Demeo DL, Campbell EJ, Barker AF, Brantly ML, Eden E, McElvaney NG, Rennard SI, Sandhaus RA, Stocks JM, Stoller JK, Strange C, Turino G, Silverman EK (2007). IL10 Polymorphisms are Associated with Airflow Obstruction in Severe alpha 1-antitrypsin Deficiency. Am J Respir Cell Mol Biol.

[B19] Novoradovsky A, Brantly ML, Waclawiw MA, Chaudhary PP, Ihara H, Qi L, Eissa NT, Barnes PM, Gabriele KM, Ehrmantraut ME, Rogliani P, Moss J (1999). Endothelial nitric oxide synthase as a potential susceptibility gene in the pathogenesis of emphysema in alpha1-antitrypsin deficiency. American journal of respiratory cell and molecular biology.

[B20] Hill AT, Campbell EJ, Bayley DL, Hill SL, Stockley RA (1999). Evidence for excessive bronchial inflammation during an acute exacerbation of chronic obstructive pulmonary disease in patients with alpha(1)-antitrypsin deficiency (PiZ). Am J Respir Crit Care Med.

[B21] Churg A, Wang X, Wang RD, Meixner SC, Pryzdial EL, Wright JL (2007). {alpha}-1-Antitrypsin Suppresses TNF{alpha} and MMP-12 Production by Cigarette Smoke-Stimulated Macrophages. Am J Respir Cell Mol Biol.

[B22] (1965). Definition and classification of chronic bronchitis for clinical and epidemiological purposes. A report to the Medical Research Council by their Committee on the Aetiology of Chronic Bronchitis. Lancet.

[B23] Dowson LJ, Guest PJ, Hill SL, Holder RL, Stockley RA (2001). High-resolution computed tomography scanning in alpha1-antitrypsin deficiency: relationship to lung function and health status. Eur Respir J.

[B24] Naidich DP, McCauley DI, Khouri NF, Stitik FP, Siegelman SS (1982). Computed tomography of bronchiectasis. Journal of computer assisted tomography.

[B25] Muller NL, Staples CA, Miller RR, Abboud RT (1988). "Density mask". An objective method to quantitate emphysema using computed tomography. Chest.

[B26] Holme J, Stockley RA (2007). Radiologic and clinical features of COPD patients with discordant pulmonary physiology: lessons from alpha1-antitrypsin deficiency. Chest.

[B27] Soejima K, Yamaguchi K, Kohda E, Takeshita K, Ito Y, Mastubara H, Oguma T, Inoue T, Okubo Y, Amakawa K, Tateno H, Shiomi T (2000). Longitudinal follow-up study of smoking-induced lung density changes by high-resolution computed tomography. American journal of respiratory and critical care medicine.

[B28] Simmonds MJ, Heward JM, Howson JM, Foxall H, Nithiyananthan R, Franklyn JA, Gough SC (2004). A systematic approach to the assessment of known TNF-alpha polymorphisms in Graves' disease. Genes Immun.

[B29] Newby PR, Roberts-Davies EL, Brand OJ, Heward JM, Franklyn JA, Gough SC, Simmonds MJ (2007). Tag SNP screening of the PDCD1 gene for association with Graves' disease. Clinical endocrinology.

[B30] Sapey E, Bayley D, Ahmad A, Newbold P, Snell N, Stockley RA (2007). Inter-relationships between inflammatory markers in stable COPD patients with bronchitis: the intra and inter patient variability. Thorax.

[B31] Barrett JC, Fry B, Maller J, Daly MJ (2005). Haploview: analysis and visualization of LD and haplotype maps. Bioinformatics (Oxford, England).

[B32] Silverman EK, Mosley JD, Rao DC, Palmer LJ, Province MA, Elston RC, Weiss ST, Campbell EJ (2001). Linkage analysis of alpha 1-antitrypsin deficiency: lessons for complex diseases. Hum Hered.

[B33] Baranova H, Perriot J, Albuisson E, Ivaschenko T, Baranov VS, Hemery B, Mouraire P, Riol N, Malet P (1997). Peculiarities of the GSTM1 0/0 genotype in French heavy smokers with various types of chronic bronchitis. Hum Genet.

[B34] Management of chronic obstructive pulmonary disease in adults in primary and secondary care. http://www.nice.org.uk/pdf/CG012_nicceguideline.pdf.

[B35] Parr DG, Guest PG, Reynolds JH, Dowson LJ, Stockley RA (2007). Prevalence and impact of bronchiectasis in alpha1-antitrypsin deficiency. Am J Respir Crit Care Med.

[B36] Rennard SI, Fogarty C, Kelsen S, Long W, Ramsdell J, Allison J, Mahler D, Saadeh C, Siler T, Snell P, Korenblat P, Smith W, Kaye M, Mandel M, Andrews C, Prabhu R, Donohue JF, Watt R, Lo KH, Schlenker-Herceg R, Barnathan ES, Murray J (2007). The safety and efficacy of infliximab in moderate to severe chronic obstructive pulmonary disease. Am J Respir Crit Care Med.

[B37] Seitz M, Wirthmuller U, Moller B, Villiger PM (2007). The -308 tumour necrosis factor-alpha gene polymorphism predicts therapeutic response to TNFalpha-blockers in rheumatoid arthritis and spondyloarthritis patients. Rheumatology (Oxford, England).

[B38] Holme J, Stockley JA, Stockley RA (2007). When should we start monitoring alpha 1 antitrypsin deficient subjects?: London..

[B39] Wright JL, Farmer SG, Churg A (2002). Synthetic serine elastase inhibitor reduces cigarette smoke-induced emphysema in guinea pigs. Am J Respir Crit Care Med.

[B40] Howard TD, Koppelman GH, Xu J, Zheng SL, Postma DS, Meyers DA, Bleecker ER (2002). Gene-gene interaction in asthma: IL4RA and IL13 in a Dutch population with asthma. Am J Hum Genet.

[B41] Bayley JP, de Rooij H, van den Elsen PJ, Huizinga TW, Verweij CL (2001). Functional analysis of linker-scan mutants spanning the -376, -308, -244, and -238 polymorphic sites of the TNF-alpha promoter. Cytokine.

[B42] Rahman P, Siannis F, Butt C, Farewell V, Peddle L, Pellett F, Gladman D (2006). TNFalpha polymorphisms and risk of psoriatic arthritis. Annals of the rheumatic diseases.

[B43] Kaluza W, Reuss E, Grossmann S, Hug R, Schopf RE, Galle PR, Maerker-Hermann E, Hoehler T (2000). Different transcriptional activity and in vitro TNF-alpha production in psoriasis patients carrying the TNF-alpha 238A promoter polymorphism. The Journal of investigative dermatology.

[B44] Demeo DL, Campbell EJ, Barker AF, Brantly ML, Eden E, McElvaney NG, Rennard SI, Sandhaus RA, Stocks JM, Stoller JK, Strange C, Turino G, Silverman EK (2008). IL10 polymorphisms are associated with airflow obstruction in severe alpha1-antitrypsin deficiency. Am J Respir Cell Mol Biol.

[B45] Vernooy JH, Kucukaycan M, Jacobs JA, Chavannes NH, Buurman WA, Dentener MA, Wouters EF (2002). Local and systemic inflammation in patients with chronic obstructive pulmonary disease: soluble tumor necrosis factor receptors are increased in sputum. Am J Respir Crit Care Med.

[B46] Sethi S, Maloney J, Grove L, Wrona C, Berenson CS (2006). Airway inflammation and bronchial bacterial colonization in chronic obstructive pulmonary disease. Am J Respir Crit Care Med.

[B47] Wilson AG, de Vries N, Pociot F, di Giovine FS, van der Putte LB, Duff GW (1993). An allelic polymorphism within the human tumor necrosis factor alpha promoter region is strongly associated with HLA A1, B8, and DR3 alleles. J Exp Med.

[B48] Colhoun HM, McKeigue PM, Davey Smith G (2003). Problems of reporting genetic associations with complex outcomes. Lancet.

[B49] Wood AM, Stockley RA (2008). Phenotypic characteristics of alpha 1 antitrypsin deficient sibling pairs show discordance: Toronto..

[B50] WTCCC (2007). Genome-wide association study of 14,000 cases of seven common diseases and 3,000 shared controls. Nature.

